# Recent Approaches to the Manufacturing of Biomimetic Multi-Phasic Scaffolds for Osteochondral Regeneration

**DOI:** 10.3390/ijms19061755

**Published:** 2018-06-13

**Authors:** Ryan Longley, Ana Marina Ferreira, Piergiorgio Gentile

**Affiliations:** School of Engineering, Newcastle University, Claremont Road, Newcastle Upon Tyne NE1 7RU, UK; ryan.longley1@newcastle.ac.uk or r.longley33@hotmail.co.uk (R.L.); Ana.Ferreira-Duarte@newcastle.ac.uk (A.M.F.)

**Keywords:** biomimetic, bi-phasic, multi-phasic, osteochondral regeneration, scaffold

## Abstract

Cartilage lesions of the knee are common disorders affecting people of all ages; as the lesion progresses, it extends to the underlying subchondral bone and an osteochondral defect appears. Osteochondral (OC) tissue compromises soft cartilage over hard subchondral bone with a calcified cartilage interface between these two tissues. Osteochondral defects can be caused by numerous factors such as trauma and arthritis. Tissue engineering offers the possibility of a sustainable and effective treatment against osteochondral defects, where the damaged tissue is replaced with a long-lasting bio-manufactured replacement tissue. This review evaluates both bi-phasic and multi-phasic scaffold-based approaches of osteochondral tissue regeneration, highlighting the importance of having an interface layer between the bone and cartilage layer. The significance of a biomimetic approach is also evidenced and shown to be more effective than the more homogenous design approach to osteochondral scaffold design. Recent scaffold materials and manufacturing techniques are reviewed as well as the current clinical progress with osteochondral regeneration scaffolds.

## 1. Introduction

Partial- and full-thickness lesions of the knee cartilage are conditions affecting people of all ages. There are many reasons for these lesions, e.g., traumatic injuries, chronic repetitive micro-trauma, and ageing [1]. Around 500,000 procedures related to cartilage disorders are performed yearly in the US alone [2]. As the cartilage lesion progresses, it extends to the underlying subchondral bone and an osteochondral defect appears. Cartilage lesions are usually not reparable due to the cartilage’s avascular nature and the consequent lack of supplementation of potentially reparative cells and bioactive factors [1,3]. Current clinical approaches for repairing cartilage include autografts, allografts, micro-fracture, autologous chondrocyte implantation and mosaicplasty. However, these methods are not completely successful due to the low quality of the new-formed tissue and the lack of restoration of the biomechanical functions [4].

Tissue engineering offers the potential of a more sustainable and effective treatment against osteochondral defects which damage articular cartilage by replacing the damaged tissue with a long-lasting bio-manufactured replacement tissue. Generally, the most common approach involves the use of a suitable biocompatible scaffold, stem cells, and a combination of bioactive molecules which include growth factor proteins [5].

This review will aim to cover a large range of scaffold-based tissue engineering approaches that will be used to regenerate bone and cartilage tissue damaged by osteochondral defects. Furthermore, this review intends to evaluate the most promising recent approaches towards scaffold design and current clinical approaches for treating osteochondral defects reported in the last 4 years. In addition, materials for osteochondral scaffolds and scaffold manufacturing techniques will be discussed.

## 2. Osteochondral Tissue Anatomy

**Figure 1:**
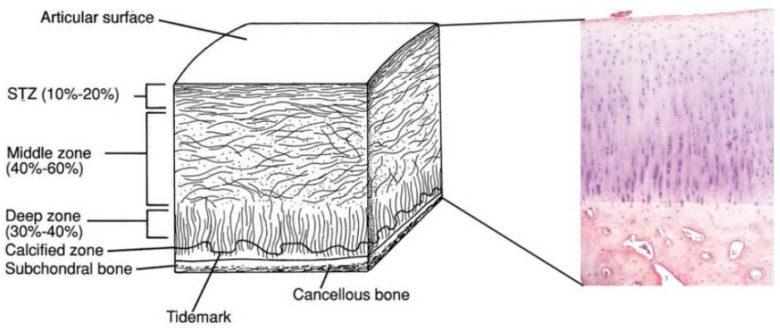
Schematic of osteochondral tissue and its components. Adapted with permission from ref [4]. Copyright 2015 by John Wiley & Sons, Inc.

At its most basic level, osteochondral (OC) tissue is based on two core components—subchondral bone and articular cartilage—and further divisions can be made within these components as follows. The subchondral bone is made of the subchondral bone plate and the calcified cartilage zone (CCZ) until the line that defines the transition into the articular cartilage, often referred to as the ‘tide mark’. The articular cartilage can further be divided into three areas: (i) the radial/deep zone, which is what the CCZ transitions into above the tide mark; (ii) the transition/middle zone, in the middle of the cartilage tissue; and (iii) the superficial tangential zone, between the joint space and the synovial fluid [6]. The osteochondral tissue is characterised by a height of around 3 mm in adults, 90% of which consists of articular cartilage, a further 5% consists of the CCZ, and the remaining 5% the subchondral bone plate. Figure 1 is a schematic representation of the OC tissue and the zones within it [4].

Particularly, the subchondral bone functions to maintain the stability of the articular cartilage above, which at the epiphysis presents characteristics that are comparable to trabecular bone: bone volume fraction in a range between 6% and 36%, a trabecular thickness of 100–190 µm, a trabecular concentration in a range between 0.61 and 2.06 trabeculae’s/mm, and a space between them in a range of 320–1670 µm [7]. The other significant region of the subchondral bone is the calcified cartilage zone, which is considered with the subchondral bone because it is a region of transition, characterised by some features of bone tissue such as alkaline phosphates and mineral deposits [8,9]. Within the calcified zone, the cartilage extracellular matrix (ECM) is mineralized and collagen type II (main component in cartilage tissue) is replaced by a distinct collagen type X. The function of the CCZ is to give a good adhesion at the interface between the subchondral bone and articular cartilage [10].

Moreover, subchondral bone is characterized by a vascular invasion of bone marrow tissue, with the presence of different types of cells [11]. Particularly, the bone marrow includes mesenchymal stromal/stem cells (hMSCs) in a very small percentage (0.002%): these have the ability to differentiate into the osteoblasts and chondrocytes. Osteoblasts are in charge of bone formation, which involves the synthesis and deposition of hydroxyapatite (HA). In cancellous bone tissue, endothelial cells are also present and they are in charge of the formation of blood vessels within the tissue [12,13].

The second component of the osteochondral tissue is the articular cartilage, which is able to distribute the load forces to the subchondral bone. The disposition and localisation of proteins, mainly collagen and proteoglycans, is optimised to support this feature [14,15]. Articular cartilage can be subdivided into three main zones, as mentioned before in this section. They consist of the radial zone, the transitional zone, and the superficial tangential zone (STZ). The STZ zone only accounts for 10–20% of the total height of the articular cartilage. The transitional and radial zones account for 80% of the total remaining height.

In articular cartilage tissue, the only cell type that is present are chondrocytes; they represent around 2% of the components in the tissue [16]. Chondrocytes are characterised by a round shape with different size and orientation throughout the cartilage depth, surrounded by collagen Type II and aggrecan, the two main ECM proteins. The cells are fairly small, approximately having a diameter of 13 µm, a surface area of 821 µm^2^ and a volume of 1748 µm^3^. These features do not vary much across the cartilage zones under normal circumstances [17]. Chondrocytes are organised into structures known as chondrons, consisting of units of single cells in the superficial and transitional zone, while in the radial zone they include around 5–8 chondrocytes [18].

## 3. Biomimetic Multi-Phasic Structure for Osteochondral Regeneration

The definition of biomimetics is “the study of the formation, structure, or function of biologically produced substances/materials and biological mechanisms and processes, especially for the purpose of synthesizing similar products by artificial mechanisms which mimic natural ones” by Merriam–Webster’s dictionary [19]. In tissue engineering, the term “biomimetics” is applied for defining the design and manufacturing of scaffolds able to mimic or imitate the biological tissue (OC tissue), providing an improved integration with the surrounding cartilage and bone tissue. Promoting osteochondral defect regeneration is a very complicated task because of the different cartilage and subchondral bone composition along with their inherent biochemical, biomechanical and biological features [20]. Therefore, it is fundamental to provide the appropriate different mechanical and biological signals for allowing the regeneration process of these two tissues [21].

The different properties presented by the tissues make it challenging to integrate and stabilise the newly formed tissue at the cartilage–bone (osteochondral) interface. Different strategies are reported in the literature, subdivided into monophasic, bi-phasic and tri-phasic scaffolds [22]. Monophasic scaffolds are not able to mimic the biological environment well and several works in the literature reported that they were inadequate to replace defective cartilage-to-bone tissue, which is characterised by anisotropic functions and structural properties [23]. For such purposes, multi-phasic scaffolds have been proposed. Several advantages of the use of multi-phasic constructs over the monophasic ones for the repair of osteochondral tissue can be described: (1) the optimisation of different types of scaffold with the addition of proper growth factors in order to mimic cartilage and bone tissue separately; and (2) the opportunity for post-assembly permits an osteogenic and chondrogenic pre-culture before the implantation in vivo [24,25]. Additionally, the multi-phasic biomimetic scaffolds may provide cells with the appropriate chemical, mechanical and biological stimuli of the tissue necessary for their proliferation and/or differentiation [26]. Multi-phasic scaffolds should also give a suitable microenvironment to direct the communications between cell/cell and cell/matrix [27].

When it comes to multi-phasic scaffolds, there tends to be two main categories depending on the physical/chemical or cellular/biological characteristics of the scaffold (Table 1). In this review we reported examples of bi-phasic and multi-phasic, acellular and biological scaffolds, where cells and biomolecules such as growth factor can be incorporated.

### 3.1. Bi-Phasic Scaffolds

A successful strategy in tissue engineering for osteochondral field includes the design of bi-phasic scaffolds with the opportunity to improve the regeneration of both cartilage and subchondral bone. The manufacturing of this type of scaffolds is achieved by independent and different processes, by which two different scaffolds for the two layers are produced and then combined. Moreover, several works in the literature report a simultaneous manufacturing process through which a unique scaffold is produced and then allowed to seed cells at the same time in both sides.

Natural polymers are largely used in the fabrication of scaffolds, due to their intrinsic biomimetic properties and great resemblance to ECM elements [28]. A bi-layered scaffold was developed by Sartori et al. [20], made from a layer of natural compound (type I atelocollagen) and another layer consisting of bioactive magnesium-doped hydroxyapatite (Mg-HA) which was co-precipitated with collagen. This design adopted a biomimetic approach by using the collagen layer to target chondral regeneration and the Mg/Ha layer to regenerate the subchondral tissue, thus mimicking the dual composition of natural osteochondral tissue. In vivo analysis of the scaffolds occurred using immunocompromised inbred nude mice and two scaffolds were implanted in each of the mice in the subcutaneous tissue after having created two pockets. The bi-layered scaffold implanted on the right side was seeded with hMSCs while the scaffold on the left was without cells. The scaffolds (size: 2 mm diameter and thickness 3 mm) were removed from the mice at 4 and 8 weeks and were evaluated using macroscopic examinations and histological analysis. The presence of rounded cells with an intense pericellular matrix was found in the chondral layer, which may be related with the hMSC differentiation in chondrocytes. On the other side, the formation of new vessels was observed in the bone layer; however, after 8 weeks, the rate of the angiogenesis process was drastically reduced. Within the same layer, the formation of bone tissue by hMSC-derived osteoblasts was observed and the presence of osteocytes was detected within this newly-formed tissue. It is also worth noting that, statistically, there was no significant difference found between the engineered and plain scaffold according to Boden’s score. This, coupled with the fact that the plain scaffolds in this study appeared colonised with organised connective tissue, could indicate that the tissue formation is influenced by the different stimuli (chemical and structural) provided by the scaffold, which is in contradiction with the literature [29,30].

On the other hand, synthetic polymers are also largely used in the scaffold manufacturing because of their low cost of processing and better functionality than natural polymers, despite the potential for an immune response or toxicity especially with the use of certain polymer combinations [31]. Synthetic bi-phasic scaffolds have been proposed by Kim et al., who proposed a composite construct, manufactured by a combination of sintering and gel-pressing technology [21]. The scaffold was based on a poly(lactide-*co*-glycolide) (PLGA)/beta-tricalcium phosphate (β-TCP) layer for mimicking bone and an elastic poly-lactide-co-caprolactone (PLCL) layer for the cartilage side. The addition of β-TCP, which is a bioactive ceramic, to the PLGA scaffold is done to have an effect on osteoconduction at the cell–material interface. The two separate scaffolds were combined together to form the bi-layered scaffold by press fitting. The final scaffold was 11 mm in length, with the PLGA/β-TCP scaffold accounting for 10 mm of the final length and the PLCL scaffold making up the remaining 1 mm. It is worth noting that the dimensions of the scaffold sections do not follow the same dimensions of natural osteochondral tissue in which the natural cartilage typically has a thickness of 2.7 mm [4].

Biological studies were completed on the scaffolds with the seeding of bone marrow mesenchymal stem cells (BMSCs) on the PLGA/β-TCP layer and chondrocytes seeded on the PLCL layer. For the in vitro study, the scaffolds were cultured in osteogenic medium for 21 days. Immunofluorescence staining was used to reveal that collagen type II was produced by the chondrocytes onto the scaffold and the cartilaginous tissue was well organised. Immunostaining also showed that after 2 days and 21 days, the chondrocytes remained within the PLCL and did not move into the osteo-layer of the scaffold. Presence of osteocalcin was observed in the PLGA/β-TCP scaffold to a limited degree after 2 days; however, this dramatically increased after 21 days. For in vivo tests, the scaffolds were implanted subcutaneously in nude mice for 6 weeks before being harvested for analysis. Histological staining showed an abundant deposit of sulphated glycosaminoglycans (GAGs) and collagen. The ECM produced by chondrocytes was formed on the PLCL layer, furthermore the top layer showed mature and well-developed cartilaginous tissue, as evidenced by chondrocytes within lacunae. Further histochemical staining revealed the presence of mineral phase, produced by osteogenic differentiated BMSCs found only in bone.

**Figure 2:**
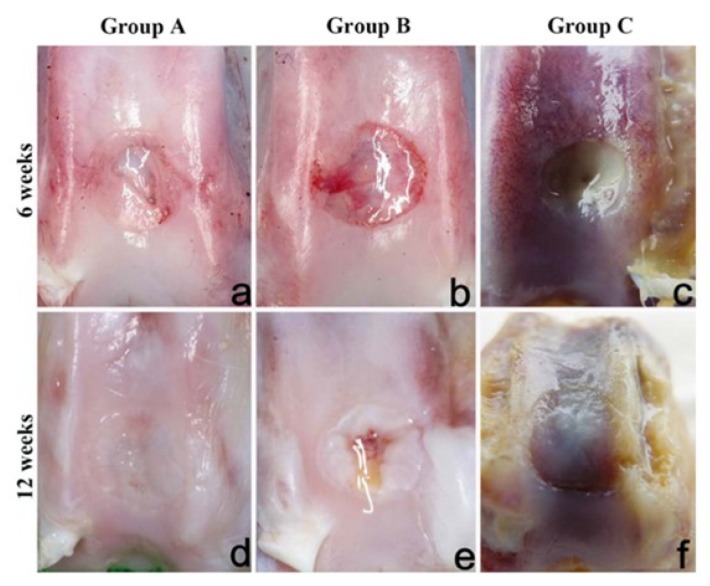
Gross appearance of the knees after implantation in vivo at 6 and 12 weeks. (**a**) At 6 weeks in group A; (**b**) At 6 weeks in group B; (**c**) At 6 weeks in group C; (**d**) At 12 weeks in group A; (**e**) At 12 weeks in group B; (**f**) At 12 weeks in group C. Adapted with permission from ref [32]. Copyright 2015 by John Wiley & Sons, Inc.

Finally, a combination of synthetic and natural polymers is a good strategy for mimicking the complex osteochondral tissue. A bioartificial bi-layered scaffold was created by Li et al. [32], consisting of a Poly-vinyl alcohol/gelatin/vanillin (PVA/Gel/V) layer combined with a nano-hydroxyapatite/polyamide-6 (*n*-HA/PA6) layer to mimic the cartilage and subchondral bone tissue respectively. The bi-layered construct was created by bonding the two distinct porous layers with a thin non-porous PVA layer. This layer prevents cross-penetration between the two scaffolds. Allogenic BMSCs were seeded onto the surface of the two layers, and they were successively chondrogenically and osteogenically induced, respectively. In vivo testing was performed by implanting the scaffold in the knees of New Zealand white rabbits. Three groups were investigated for the experiment: (1) the defect in one knee was treated with BMSC-seeded bi-layered implant (Group A); (2) the defect in the opposite knee of the same test subject treated just the bi-layered scaffold (Group B); the remaining rabbits were used as controls and the defects in both of their knees were left untreated (Group C). The OC tissues were evaluated both histologically and macroscopically after 6 and 12 weeks from the implant. The gross morphology of the knees was assessed and the appearance of each group can be seen in (Figure 2) below. After 6 weeks of implantation, Group B defects showed the formation of newly smooth white tissue; however, this new tissue was not observed in the defects’ middle region (Figure 2b). In contrast, Group A exhibited defects that were mainly filled with an opaque tissue (Figure 2a), and after 12 weeks post-operation they fully filled with new-formed cartilage-like tissue, similar in texture and colour to the close articular cartilage (Figure 2d). At 12 weeks, Group B defects presented a translucent and white tissue (Figure 2e).

The last two reported works have investigated bi-layered scaffolds using histology as part of their evaluation for in vivo experiments performed on the scaffolds; therefore, we may assume that the biological results obtained in these investigations could be directly compared and used to help further new developed osteochondral scaffolds. Unfortunately, this is not the case due to multiple factors which make comparisons between each investigation more difficult. One of the issues is represented by the in vivo tests, where the scaffolds were implanted “subcutaneously” or under the skin. This does not represent accurately the typical site where an osteochondral scaffold would be implanted in clinical application. The implantation site in these investigations also did not require the scaffold to bear any load. The issues highlighted here are due to the lack of any sort of regulations or guidelines that have been universally accepted by the researches working within the field of osteochondral tissue engineering. This observation was also recognised by Izadifar [3]. However, a preliminary in vivo evaluation by using subcutaneous implants can give an opportunity to study the spontaneous behaviour of new biomaterials/scaffolds in inducing the correct cell differentiation (e.g., chondroinduction, osteoinduction, etc.) before the following implant in OC defects.

### 3.2. Tri-Phasic/Multi-Phasic Scaffolds

Recently, the interest in OC regeneration to manufacture not only bi-phasic but multi-phasic scaffolds in order to mimic better the native OC tissue has been dramatically increasing. [33,34]. One major focus of these multi-phasic scaffolds is the interface between the cartilage and bone tissue, or the layer of the scaffold that would mimic what the CCZ found in natural osteochondral tissue. Understanding the transition between vascular and mineralized bone and the un-vascular and un-mineralized cartilage is crucial for the correct design of the cartilage–bone interface [35,36]. To date, the design and formation of a stable interface between cartilage and subchondral bone in a multi-phasic scaffold remains a significant challenge [33]. Some recent studies and review articles have showcased various efforts in interface design for osteochondral scaffolds. 

The combination of conventional and unconventional methods for the manufacturing of scaffolds is a suitable strategy to create and mimic the morphology and composition of the native osteochondral tissue. Jeon et al. [37] showcased a novel multi-phasic scaffold that consisted of a bi-layered 2% alginate which contained superficial chondrocyte in the upper layer and middle-deep chondrocyte in the lower layer, which made up the cartilage portion of the scaffold. This bi-layered scaffold was joined to the poly(ε-caprolactone) (PCL) scaffold made with fused deposition modelling (FDM) which is also joined to an electrospun PCL layer, both seeded with osteoblasts. The PCL scaffold represents the cartilage–bone interface and the electrospun scaffold represents the subchondral bone. The investigation also included a novel approach to in vivo testing in which the scaffolds were encased within a cylindrical bovine osteochondral plug. Batches of eight multi-phasic scaffold/bovine plugs were then implanted subcutaneously for 12 weeks within immunocompromised rats.

In this experiment, the use of bi-layered cartilage gel did not summarise the compartmental structure of the native cartilage, nor zone-specific distribution of glycosaminoglycan. The bone section of the scaffold lacked bone ingrowth and mineralisation, most likely due to insufficient vascularisation. Although unsuccessful biological results were obtained, this work reported a well-designed and new method for the division of the scaffold into different areas for resembling the gradient behaviour of the OC interface. However, a limitation on this approach may be represented by the construct size (8 mm long scaffold) with problems in integrating within the surrounding tissue, likely due to the lack of associated vasculature. The results of this study are difficult to compare to others, because of the novel approach of encasing the scaffold within a bovine osteochondral plug and what possible effects they could have had on the overall performance of the scaffold.

**Figure 3:**
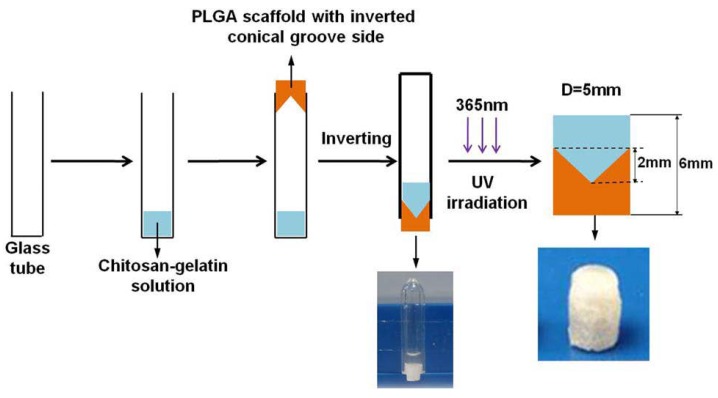
Scheme of the preparation of the conically graded chitosan-gelatin hydrogel/poly(lactide-*co*-glycolide) (PLGA) scaffold. Adapted with permission from ref [38]. Copyright 2014 by John Wiley & Sons, Inc.

In a further study, Han et al. [38] designed a graded construct for mimicking and repairing the cartilage–bone interface tissue via incorporation of specific growth factors into the multi-phasic construct. Particularly, they obtained a conically graded transition from a chitosan and gelatin-based hydrogel, loaded with high dose of Transforming growth factor (TGF)-β1 (simulating cartilage layer) to a PLGA porous scaffold loaded with high dose of Bone morphogenetic protein 2 (BMP-2) (simulating bone layer) (Figure 3).

As reported in this work, the direct injection of biomolecules (i.e., growth factors) for treating diseases requires high doses, compromising their effectiveness and in vivo life. Therefore, to overcome these issues, the authors used a suitable delivery system for a controlled, local and prolonged growth factor release via encapsulation into PLGA microspheres. In vivo testing was carried out by using New Zealand white rabbits for 2 months, and it was observed that the incorporation of the growth factor-loaded micro-particles in the multi-layered construct promoted the osteochondral regeneration dramatically, where the defects were filled with new chondral and subchondral bone tissues with a similar morphology to the normal tissue.

Particularly, TGF-β1 influenced the MSC proliferation and differentiation with ECM production [39], which led to the formation of a new hyaline-like cartilage tissue. On the other hand, BMP-2 stimulated chondrogenesis and osteogenesis differentiations of MSCs [40] and promoted alkaline phosphatase activity.

**Figure 4:**
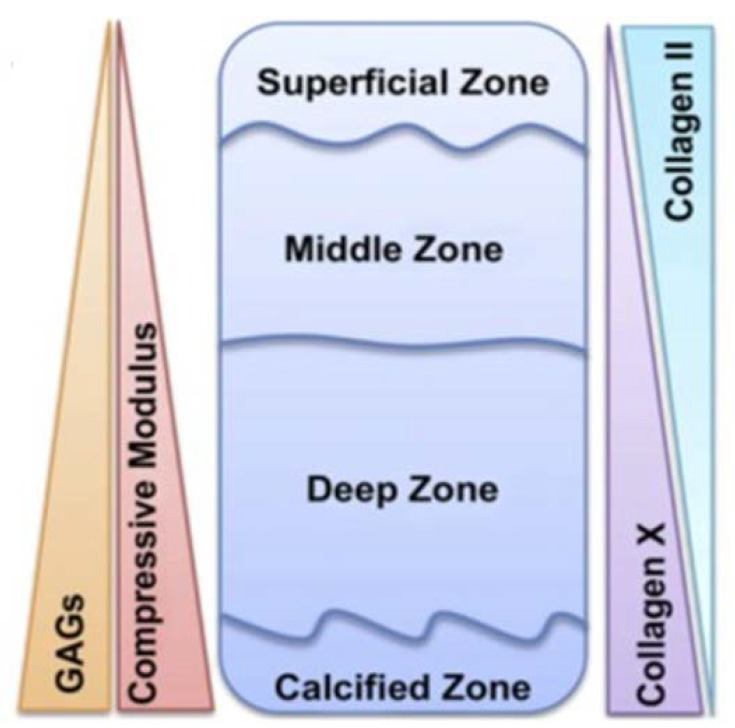
Schematic of articular cartilage anatomy which illustrates how the GAG content, collagen X and compressive modulus increase from the superficial to the deep zones of articular cartilage. Collagen II is also shown too decrease in content from the superficial to the deep zones of articular cartilage. Adapted with permission from ref [41]. Copyright 2011 by Elsevier, Inc.

Another strategy for regenerating osteochondral defects is represented by the use of hydrogels, which have received a remarkable interest as suitable candidates for tissue engineering due to their unique compositional and structural similarities to ECM, in addition to their desirable framework for cellular proliferation and survival. As reported by Nguyen et al., natural articular cartilage composition can be subdivided into four distinct zones (Figure 4) [41]; therefore, a biomimetic multi-phasic scaffold could be designed in different layers that direct marrow-derived stem cells into varying cell phenotypes that resemble cells from the superficial, transitional, deep and calcified zones of articular cartilage.

**Figure 5:**
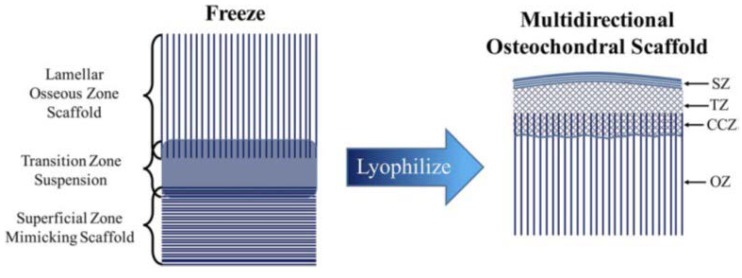
Diagram of the lyophilisation process used to fabricate the final osteochondral scaffold. Scaffold contains superficial (SZ), transition (TZ), calcified cartilage (CCZ), and osseous zones (OZ). Adapted with permission from ref [42]. Copyright 2011 by John Wiley & Sons, Inc.

This strategy has been applied in the manufacturing of multi-phasic scaffolds by Clearfield et al. in 2018 [42]. In their work they demonstrated that a biomimetic multi-layered construct may imitate better the zonal structure of osteochondral tissue. It consisted of three distinct layers, the superficial or cartilage layer fabricated by unidirectional freeze casting collagen-hyaluronic acid, the osseous layer fabricated using the unidirectional freeze casting of collagen-hydroxyapatite-containing suspensions instead, and the transition zone or the CCZ manufactured by lyophilisation bonding process for joining the two distinct scaffolds (described above) together as shown in Figure 5. The final assembly is a fully integrated scaffold fabricated from three independent material compositions.

All these studies highlight the benefits of a biomimetic design approach towards osteochondral tissue engineering and show how having a construct that mimics the natural structure of osteochondral tissue will improve the performance of a tissue engineered approach that uses a scaffold (more in vivo animal tests of multi-phasic scaffolds are reported in Table 2). Current studies show tri-phasic/multi-phasic scaffolds as promising approach to develop biomimetic osteochondral constructs in order to mimic the OC interface. The recent awareness of the significance the interface between the chondral and subchondral bone plays in OC tissue engineering has started to be carefully considered. This point has been described as “critical for graft integration and for establishing long-term functionality” [43,44]. Hunziker et al. demonstrated that a physical barrier is essential for maintaining the stability of the neo-cartilage formed post repair and would prevent unwanted bony ingrowth. Therefore, the next stage in osteochondral scaffold design must take into consideration the regeneration of the osteochondral interface [45].

## 4. Clinical Progress and Insight into the Still Open Challenges

Scaffolds that treat defects in the osteochondral tissue, such as cartilage lesions or osteoarthritis, are currently not an available option for patients in typical medical care. Very few scaffold designs have even made it to clinical trials. This paragraph will review the current state of osteochondral scaffolds in clinic and provide an insight into the challenges that are still faced in the field.

The tissue engineering strategy of manufacturing homogenous scaffolds for articular cartilage has failed to reach prevalent clinical efficiency due to the fact that bulk properties of these substitutes do not imitate properly the functions of native tissue [49]. More success has been achieved with scaffolds that resemble osteochondral tissue more accurately, namely multi-layered or hierarchical tissue engineering approaches. Up to 2017, there are currently only three scaffold designs of this nature that have reached the phase of clinical trials (Table 3).

The TruFit™ CB (Cartilage/Bone) is a bi-phasic resorbable implant consisting of semi-porous PLGA–Poly-glycolic acid (PGA) (75:25) and calcium phosphate [50]. The implant is designed to replicate the mechanical properties of the articular cartilage (PLGA-PGA) and the bone (calcium phosphate) aspects of osteochondral tissue. The scaffolds are attached to each other with a small amount of solvent after individual preparation. One clinical study described a slow improvement of the injured site but issues of delayed integration was also reported [51]. Another clinical study by Barber et al. showed much less favourable results. CT scans performed on nine patients implanted with the TruFit™ were done at intervals between 2 and 63 months.

They revealed virtually no evidence of bone ingrowth, osteoconductivity, or ossification [52]. In Verhaegen et al.’s recent review, it was observed (through magnetic resonance imaging (MRI)) that there was a consistent swelling associated with all TruFit™ plugs [53]. This could be attributed to the delayed integration of the implant seen in multiple studies. The implant did also demonstrate stable cartilage-like repair at 6–12 months in most studies. Long-term results (more than 2 years) were also questionable, as highlighted by how the plugs were used to treat patellar osteochondral lesions. The failure of the implant was attributed to delayed subchondral lamina formation [50]. From the clinical studies performed on the TruFit™ scaffold, it is clear that it requires further development in order to treat osteochondral injuries more effectively and consistently. Judging by the poor integration with the surrounding tissue, it would likely benefit the scaffold if the design more closely mimicked the surrounding osteochondral tissue rather than the two-layered approach of just cartilage and bone tissue. The addition of an anti-inflammatory agent could also be incorporated into the construct in order to combat the issue of inflammation that was highlighted by Kang et al. [54] and Fini et al. [55].

The second bi-phasic scaffold currently in clinical trials is known as the “Agili-C™ CartiHeal”. The bone layer of the scaffold consists of crystalline aragonite (calcium carbonate based) and the cartilage compartment is made of hyaluronic acid [4]. Before reaching clinical trials, the scaffold was optimised in a goat model, where different construct designs were tested. The results indicated that scaffolds with two drilled phases performed better in the animal model after 6 months so this design was moved forward into clinical trials. For clinical trials, the scaffold was implanted in a 47 year-old nonprofessional sportsman resulting in the successful treatment of a femoral condyle osteochondral lesion of 2 cm^2^. After 18 months, the patient returned to his preinjury sporting activity. At 24 months, MRI analysis showed promising results in terms of articular cartilage restoration [4]. Although initial clinical results look promising, compared to the other two osteochondral scaffolds, in the clinic this scaffold has not undergone vigorous clinical testing. To add to this, the first clinical test was performed on one patient only. The activity levels of the patient could also factor into the good results obtained in the clinic. Several studies have hypothesised the importance of mechanical stimuli on the scaffold after implantation and how this can stimulate cells to differentiate and grow [56]. With the patient being a sportsman, it was likely that he would have exercised or performed physiotherapy to some degree during the trial which could have aided the scaffold’s performance because of the reasons outlined above. In other clinical trials for other scaffolds this might have not taken place with particular patients. In order to determine if the scaffold can treat osteochondral defects effectively more clinical trials must be conducted. 

The third and final scaffold currently undergoing clinical trials is the tri-phasic Maioregen™, incorporating a biomimetic design that attempts to resemble the structure of osteochondral tissue more closely [57]. The cartilage layer consists of equine type I collagen and has a thickness of 2 mm. The intermediate (tidemark like) layer consists of a combination of type I collagen (60% of weight) and magnesium-hydroxyapatite (Mg-HA) (40% of weight). The lower layer consists of a mineralised blend of type I collagen (30% of weight) and Mg-HA (70% of weight) in order to reproduce the subchondral bone tissue. Using the nucleation of HA nanocrystals onto self-assembled collagen fibers, a biomaterial was generated that could be graded in order to mimic the hierarchical layered structure of osteochondral tissue while also resembling the composition of the ECM’s of cartilage and bone tissues.

Clinical studies were performed by Kon et al. in which the scaffold was implanted into the knees of patients who were suffering from chondral defects (average defect size: 3.2 ± 2.0 cm^2^) [58]. The trial evaluated 30 patients (under 60 years old), 15 of these were implanted with the Maioregen™ scaffold. Results were reported for 13 of the 15 patients as early detachment of the implant was found in two of these patients. The outcome of this study was promising, with patients showing an increase in their normal daily activities as indicated by the subjective International Knee Documentation Committee (IKDC) score improvements. After 6 months, subchondral bone formation was observed without the presence of biomaterial with histological analysis, indicating complete resorption of the biomaterial after 6 months. The cartilage tissue appeared not only repaired but engaged in an ongoing maturation process. These promising results led to a longer follow up with randomised studies. The same authors went on to evaluate 28 patients over the period of 2–5 years after surgery. At 3 years post-surgery, the IKDC evaluation score showed significant improvement compared to the score achieved by the control group. MRI results obtained at this time showed complete repair and filling of the defect in 66.7% of cases. At 5 years follow up, MRI evaluation revealed significant improvement in both cartilage and subchondral bone status.

These clinical results obtained by the Maioregen™ highlights the advantages of having a hierarchical graded structure that mimics more closely the natural structure of the OC tissue. It also shows that using biomaterials that resemble the ECM of osteochondral tissue will improve the scaffold’s performance. However, in a recent work Christensen et al. observed opposite and negative outcomes, resulting in an incomplete cartilage repair and poor subchondral bone repair at 1 and 2.5-year follow-up after treating osteochondral defects in the ankle and knee joint with MaioRegen^®^ scaffold [59].

## 5. Conclusions

From the vast array of studies that have been carried out over the past few years, it is clear that scaffold-based tissue engineered approaches offer a lot of promise towards the treatment of OC defects. The advancements throughout the field are highlighted by the relatively short transitional period it has taken from using monophasic scaffolds to using multi-phasic scaffolds in an attempt to regenerate OC tissue. This specific example is encompassed by the larger transition of using a “biomimetic” approach towards osteochondral tissue engineering. This is highlighted with respect to morphological and structural advancements in scaffold design seen throughout the research. The importance of a scaffold’s ability to mimic the ECM on a molecular level is another priority that all future approaches must take into account.

The need for a regulatory system for scaffold in vitro/in vivo test results is made apparent by the difficulty in comparing scaffolds’ results with each other from different scientific papers. By having universal grading criteria and a specific set of tests with controlled parameters, it would become possible to compare results between different scaffold designs and help establish which particular features of OC scaffolds optimise performance. Clinical results from the current OC scaffolds indicate that a tri-phasic approach offers the most promising results with patients and their conditions. However, all scaffolds that have been tested in clinic lack appropriate mechanical properties to safely support an OC defect when under in vivo biomechanical forces. To overcome the issues that still prevent OC scaffolds from reaching good long-term clinical standards with a significant majority of patients will require a continued collaborative effort between biomedical engineers, material scientists, clinicians and developmental biologists. Finally, the possibility of using the additive manufacturing technique with synthetic polymers offers the potential advantages of intricate scaffold design control of particular parameters and consistent reproduction of scaffolds that could be designed to fit each patient personally.

## Figures and Tables

**Table 1:** Osteochondral phasic scaffold design strategies.

Type of scaffold		Properties	
Monophasic Scaffold	Acellular	- One material - Homogenous porosity	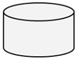
	Biological	- One cell phenotype	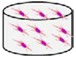
Bi-phasic Scaffold	Acellular	- Two materials Or - One material with two phases/layers with different porosity	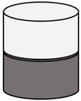
	Biological	- Two different cell phenotype Or - One cell type with two pre-differentiation states or two biological micro-environments	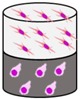
Tri-phasic or Multi-phasic Scaffolds	Acellular	- Three or more materials Or - One material with three or more phases/layers with different porosity	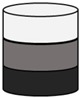
	Biological	- Three or more cell phenotype Or - One cell type with three or more pre-differentiation states or three or more biological micro-environments	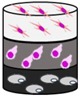

**Table 2:** Summary table on the most recent in vivo implant of bi-phasic and multi-phasic osteochondral scaffolds.

	Scaffold Type and Composition	Manufacturing Process	In Vitro and/or In Vivo Analysis	Reference
Bi-phasic	Acellular scaffold Top: Alginate + TGF-β1-loaded microspheres Bottom: PLGA + BMP2-loaded microspheres	Top: Freeze-drying Bottom: Gas foaming	In vivo implant in cylindrical osteochondral defects (diameter 4.5 mm, deep 4 mm) in adult male New Zealand rabbits for 24 weeks:­ - The functionalised scaffolds exhibited a high degree of repair, with the cartilage layer being thicker than the normal adjacent cartilage in the center but not at the defect borders - No apparent signs of osteochondral defect repair in untreated or low-dose growth factor (GF)-loaded scaffolds (scores 0–4), while high GF dose-loaded scaffolds showed clear signs of repair (scores 12–17)	Reyes et al. 2014 [46]
	Acellular scaffold Top: silk fibroin Bottom: silk fibroin + nano calcium phosphate powder	Salt leaching + freeze-drying	In vivo implant in cylindrical osteochondral defects (diameter 4.5 mm, deep 5 mm) in New Zealand White rabbits (9–11 weeks old) for 4 weeks:­ - Good integration with a layer of connective tissue adhered on the entire surface of the scaffolds without signs of infection or acute inflammation - Less void space and more regular morphology into the defect filled with the scaffold compared with the defect control (30% filled with new bone)	Yan et al. 2015 [47]
	Cellular scaffold (BMSCs seeded on the construct for 3 days before implant) Top: PLCL Bottom: PLGA/ β-TCP	Top: sintering Bottom: gel pressing	In vivo implant in subcutaneous implantation in nude mice (7 week old) for 6 weeks: - Mature and well-developed cartilaginous tissue, as evidenced by chondrocytes within lacunae - Presence of calcium phosphates in the bone layer	Kim et al. 2015 [21]
	Cellular scaffold (MSCs seeded on the construct before implant) Top: PVA/Gel/V Bottom: n-HA/PA6	Freezing-thawing	In vivo implant osteochondral defects (diameter 4 mm, deep 6 mm) in New Zealand rabbits for 12 weeks: - BMSC-loaded constructs exhibited defects that were mainly filled with an opaque tissue (new cartilage-like tissue) - Acellular construct presented translucent and white tissue	Li et al. 2015 [32]
	Cellular scaffold (hMSCs seeded on the construct before implant) Top: Type I atelocollagen Bottom: Mg-doped HA	Freeze-drying	In vivo subcutaneous implant in mice for 8 weeks: - Scaffold layers appeared well integrated without interruptions or cells agglomerates at the interface between the two layers - Neoangiogenesis seemed to be less prominent in comparison to the one observed at 4 weeks - In chondral layer, there are spherical cells, surrounded by lacuna closely resembling mature chondrocytes, while in the bone layer the formation of bone tissue by hMSCc-derived osteoblasts was detected	Sartori et al. 2017 [20]
	Acellular scaffold Top: type I and II collagen and hyaluronic acid Middle: type I and II collagen and HA Bottom: HydroxyColl^TM^, composed of type I collagen and HA, commercialised by SurgaColl Technologies	Freeze-drying	In vivo implant in cylindrical osteochondral defects (diameter 3 mm, deep 5 mm) in New Zealand White rabbits (9 months old) for 12 weeks: - Quantification of bone formation from 3D Micro-CT reconstructions in the multi-layered scaffold group (0.401 ± 0.0523) was found to be significantly greater than that in the empty defect group (0.351 ± 0.0309) - Presence of proteoglycans and cartilage were observed in the multi-layered scaffold group by histological analysis whereas fibrous tissue was observed in the empty defect group	Levingstone et al. 2016 [48]
Multi-phasic	Cellular scaffold (MSCs seeded on the construct before implant) Cartilage layer: different layers of CS- glycidyl methacrylate( GMA) and Gel-GMA loaded with TGF-β1 Bone layer: PLGA loaded with BMP-2	Cartilage layer: hydrogels via UV polymerisation Bone layer: separation/particle leaching method	In vivo implant in cylindrical osteochondral defects (diameter 5 mm, deep 6 mm) in New Zealand White rabbits for 8 weeks: - TGF-β1 influenced the MSC proliferation and differentiation with ECM production, that led to the formation of a new hyaline-like cartilage tissue - BMP-2 stimulated chondrogenesis and osteogenesis differentiations of MSCs and promoted alkaline phosphatase activity.	Han et al. 2014 [38]
	Cellular scaffold Cartilage layer: Top: alginate with superficial chondrocytes Bottom: alginate with middle-deep chondrocytes Bone layer: PCL with osteoblasts	Cartilage layer: ionic crosslinked alginate with CaCl_2_Bone layer: FDM	Ectopic osteochondral model. Bovine osteochondral cores prepared from bovine knees were filled with the construct prior to subcutaneous implantation in nude mice (8 week old) for 12 weeks: - Good integration of all the layers - Limited mineralisation in the PCL compartment with or without the pre-seeded osteoblasts - Limited blood vessel network within the osseous construct while there were many blood vessels found within the bovine bone	Jeon et al. 2018 [37]

hMSCs: mesenchymal stromal/stem cells; HA: Hydroxyapatite BMSC: bone marrow mesenchymal stem cells; FDM: fused deposition modelling.

**Table 3:** Summary table reporting the details of the currently three scaffold designs that have reached the phase of clinical trials.

Scaffold	Name and Sponsor	Materials	Plug Size and Depth	References
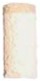	TruFit CB™, Smith & Nephew	Bi-phasic implant consisting of semi-porous PLGA-PGA (75:25) and Calcium-phosphate	Diam 5–11 mm, 18 mm	[50,51,52,53,54]
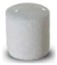	Agili-C™, CartiHeal Ltd.	Crystalline aragonite (calcium carbonate based) and hyaluronic acid	Diam 6–18 mm,15 or 20 mm	[4,55].
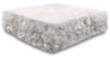	Maioregen™, Finceramica	Cartilage layer: equine type I collagenTidemark like layer: type I collagen (60%), Mg-HA (40%) Lower layer: mineralised blend of type I collagen (30%), Mg-HA (70%)	35 × 35 mm, 6 mm (±2 mm due to the swelling)	[56,57,58]

## Abbreviations

ALP	Alkaline phosphate activity
BMSCs	Bone marrow mesenchymal stem
β-TCP	Beta-tricalcium phosphate
CCZ	Calcified cartilage zone
ECM	Extracellular matrix
Gel	Gelatin
HA	Hydroxyapatite
hMSCs	Human mesenchymal stromal/stem cells
IKDC	International Knee Documentation Committee
Mg-HA	Magnesium-doped hydroxyapatite
MRI	Magnetic Resonance Imaging
n-HA	Nano-Hydroxyapatite
OC	Osteochondral
PA6	Polyamide-6
PEG	Polyethylene glycol
PEG-Da	Polyethylene glycol diacrylate
PGA	Poly-glycolic acid
PLGA	Poly(lactide-*co*-glycolide)
PLCL	Poly-lactide-*co*-caprolactone
PVA	Poly-vinyl alcohol
STZ	Superficial tangential zone
V	Vanillin
